# Moral Disengagement as a Moderating Factor in the Relationship between the Perception of Dating Violence and Victimization

**DOI:** 10.3390/ijerph17145164

**Published:** 2020-07-17

**Authors:** Isabel Cuadrado-Gordillo, Inmaculada Fernández-Antelo, Guadalupe Martín-Mora Parra

**Affiliations:** Department of Psychology and Anthropology, University of Extremadura, 06071 Badajoz, Spain; iferant@unex.es (I.F.-A.); guadammp@gmail.com (G.M.-M.P.)

**Keywords:** dating violence, adolescents, victimization, moral disengagement, acceptance of violence, moderating effects

## Abstract

There have been studies establishing the relationship between moral disengagement and aggressiveness in various contexts, especially in the role of the aggressor. Few, however, have analyzed moral disengagement’s mediating role in the phenomenon of teenage dating violence, taking into account how these mechanisms affect the victims’ perception of themselves as fearful, trapped, or mistreated in a dating relationship. This study analyzes the relationship between moral disengagement, the acceptance of violence, and how the victims of this type of abuse perceive victimization. The participants were 2577 adolescents between the ages of 14 and 18. They completed two questionnaires that addressed teenage dating violence and moral disengagement. To study the relationship between the variables, factorial, structural, correlation, and hierarchical multiple regression analyses were performed to construct the perceptual structure of victimization. The analyses showed moral disengagement and the acceptance of violence, as well as their interaction, to have a mediating and moderating influence by modifying the perception of victimization. The victims’ levels of moral disengagement explained their acceptance of the violence and their inability to recognize abuse. Finally, these results may be a key element in the design of psychological interventions aimed at minimizing the use of moral disengagement and the acceptance of violence in situations involving aggression in teenage dating.

## 1. Introduction

Violence in teenage dating is classified as a phenomenon of an aggressive nature that includes physical, psychological, sexual, and/or emotional abuse taking place within a relationship at ages from early adolescence to the beginning of adulthood [1]. The presence of violence in teenage dating is considered to be one of the main predictors of gender violence in adulthood [2].

In recent times, the rise in violence in teenage dating has become a public health problem worldwide. Consequently, there has been exponential growth in research aimed at tackling this phenomenon [3,4]. It has not been easy, however, to find any explanation for the growth of this type of abusive behavior, mainly due to its multi-causal nature. While there has been in-depth research on the determining role of certain variables, sexism for example, in aggression and in the acceptance of violence [5,6,7], there has been less attention paid to other factors, such as moral disengagement (fundamentally in its mediating function with variables involved in the victimization process).

### 1.1. Dating Violence: Perception of Aggressive Behavior from the Victims’ Point of View

During adolescence, the development of the first dating relationships is of vital importance since they will define future relationships, confirming or rejecting the preconceptions that adolescents have regarding dating [8]. Maintaining or altering the said perceptions can lead to certain behaviors being seen as normal, or even desirable (jealousy, control, etc.), and can modify how adolescents classify what behaviors are or are not abusive [9]. Such modification of perceptions runs the risk of normalizing aggressive behavior. A normalized perception of abuse becomes a risk factor for the transmission of violence from one relationship to another, and even from one generation to another [10]. In this sense, some researchers have noted that, in those teenagers with a high level of acceptance of dating violence, peer aggression and delinquency are significant predictors of recurrent or chronic abuse in a new relationship [11].

The normalized perception of violent behavior and abuse by adolescent victims has been explained through various factors. In this sense, some research has pointed to how society tends to consider people with violent attitudes and behaviors as having an attractive and exciting character. These types of message, transmitted through the media, make adolescent girls socialize in the imbalance of power that exists between men and women, and make them choose aggressive partners whom they characterize as being the perfect prototype [12]. On the other hand, the perception they have of relationships that do not have these characteristics is that they are less emotionally touching [13,14], and therefore less attractive. For this reason, and especially during adolescence, dating relationships are frequently idealized, creating a hard to attain utopian archetype in which the victims perceive that anything goes in pursuit of the ideal they have created, and around which practically any behavior can be valid, since love can do everything [15].

It must be taken into account how gender stereotypes and roles influence the perception of the behavior that both men and women must have when dating. The roles traditionally associated with the feminine and the masculine can lead to an altered perception of the behavior that each member of the couple has within the relationship. Thus, when violence coincides with gender stereotypes, it is perceived with less seriousness [16].

### 1.2. Influence of Moral Disengagement on Violent Behavior in Teenage Dating

From a cognitive point of view, there are factors that may have equal weight in relation to adolescents’ perception of dating violence. Specifically, their morality and the mechanisms of moral disengagement that they use could affect the view that both aggressors and victims have of themselves.

The concept of moral disengagement can be defined by referring to the mechanisms people use as buffers between their individual moral principles and their actual behavior [17]. Thus, it is especially useful not only to try to legitimize behavior that is violent or goes against the established social and personal morality, but also to understand aggressive behavior as being a legitimate way to pursue and achieve personal goals [18]. To implement this process of legitimization, moral disengagement makes use of eight mechanisms that act on different loci in people’s self-regulation system. These mechanisms are: euphemistic language, moral justification, advantageous comparison, displacement of responsibility, diffusion of responsibility, distortion of consequences, attribution of blame, and dehumanization [17,19].

There has been little research on violence in teenage dating with exploration of the possible implications that moral disengagement mechanisms might have on this phenomenon. The focus of the few studies that have been carried out has tended to be on the figure of the aggressor. The results point to the existence of certain relationships between the moral deficiencies of the aggressors and the violent acts they commit, especially with regard to sexual assaults. Bandura [20] originally argued that moral disengagement mechanisms could be found incorporated into the mythology of rape, exonerating the aggressors and blaming the victims. This finding was later confirmed and expanded on in studies which noted that, in adolescent dating relationships, sexual aggressions are related to lower levels of guilt and shame [21]. Diverse research studies have found relationships between the eight mechanisms of moral disengagement and the aggressors’ violent behavior. For example, Tata [22] found that the use of a sense of humor is a form of employing euphemistic language that allows sex offenders to deny their responsibility by labeling their behavior as jokes of no importance. In this regard, other authors have noted how the diffusion of responsibility is used to downplay the act of sexual harassment perpetrated when such acts are carried out in groups of peers. With this, people minimize their personal involvement by sharing the blame out over the other members of the group [23].

There have been even fewer victim-focused studies addressing the determining role that moral disengagement mechanisms might play in victimization. Nevertheless, some authors have noted how learning and using the mechanisms of moral disengagement are a way to rationalize aggressive behavior, and this implies that the victims come to learn that aggression is an appropriate way to respond in disagreements [24]. It is usual for this same phenomenon to appear in children who have suffered abuse during infancy, with their using it as a way to justify, reconstruct, or rationalize the violence they have suffered [25]. Furthermore, it has been found that adolescents who have experienced a higher level of rejection are precisely those who end up developing higher levels of moral disengagement relative to those who have not undergone this type of situation [26].

The cognitive rationalization implied by the use of moral disengagement in victimization has been examined in various violent phenomena, including bullying and cyberbullying. Some researchers have found that victims tend to develop empathy towards their aggressors, and use moral justification as a way to protect their self-esteem [27]. Others have also linked victims’ use of moral disengagement with their justification of both the aggressors’ behavior and their own lack of activity when facing the abuse [28,29]. Cuadrado and Fernández [30] indicated that moral disengagement mediates the relationship between victimization and the perception of the aggression since victims use it in order to minimize the abuse suffered, thereby avoiding the need to ask for help in situations of risk or danger.

Finally, although research focused on violence in teenage dating is scarce, the results commented on above seem also to be applicable to this phenomenon since recent research, has found that victims may use moral justification when they experience violence during their relationship, although this was only found in the case of the youngest subjects of their sample—those of ages from 16 to 18 [2]. Likewise, the results of that study showed that dehumanization is a moderator of the relationship between moral disengagement and victimization in a dating relationship. This indicates that those people with high scores in the use of the two mechanisms, i.e., dehumanization and moral justification, are those who report having suffered a greater number of aggressions.

Taking into account the few research studies that have analyzed the role that moral disengagement may play for the victims, it is necessary to analyze this relationship in greater depth in order to understand what are the factors that may be perpetuating victimization, even leading to the victims not seeing themselves as such, which makes detection and intervention difficult. In this sense, the moral disengagement mechanisms function in adolescents by deactivating their self-regulation moral mechanisms [31]. Applied to the phenomenon of dating violence, these facts could be contributing to the perception that victims form of their relationships. Therefore, the specific objective of the present study was to explore the possible mediating role of moral disengagement in the relationship between self-perception of the abuse suffered and victimization. To respond to these objectives, we formulated the following hypotheses:

**Hypothesis** **1 (H1).** *Moral disengagement will exert a mediating effect on the relationship between the perception of dating violence and victimization.*

**Hypothesis** **2 (H2).** *Acceptance will moderate the relationship between the perception of dating violence and victimization via moral disengagement.*

## 2. Materials and Methods 

### 2.1. Participants

The sample consisted of a total of 2577 adolescents between the ages of 14 and 18 (M = 16.17; SD = 1.20). These participants were selected following an approximately proportional stratified procedure that included different Spanish secondary education schools. Both urban and rural areas were included so as to cover populations with different sociocultural characteristics. In the urban areas, schools were selected in residential areas in which the purchasing level is medium to high, and in more modest neighborhoods in which people live who normally work in low-skilled trades with a medium to low purchasing power. In the rural areas selected, the family income was lower than the regional average, and approximately half of the participants’ parents did not have a university education.

### 2.2. Instruments

For the development of this research, two questionnaires were used:

- *Dating Violence Questionnaire* (*Cuestionario de Violencia de Novios, CUVINO*) [32]. This questionnaire consists of a total of 61 items grouped into three thematic blocks. The first of these blocks is subdivided into two: one is designed to measure the frequency of the violence, and the other, the degree of tolerance that this behavior provokes or might provoke in adolescents. In both cases, a 5-point Likert scale is used, but with different anchors—in the first case (frequency) from “Never” to “Almost always”, and in the second (tolerance) from “None” to “A lot”. The pairs of scores obtained in this first block can be grouped into a total of 8 sub-scales: detachment (“Is a good student, but is always late at meetings, does not fulfill his/her promises, and is irresponsible”), humiliation (“Ridicules your way of expressing yourself”), sexual (“You feel forced to perform certain sexual acts”), sexual (“You feel forced to perform certain sexual acts”), coercion (“Threatens to commit suicide or hurt himself/herself if you leave him/her”), physical (“Has thrown blunt instruments at you”), gender (“Has ridiculed or insulted women or men as a group”), emotional punishment (“Refuses to give you support or affection as punishment”), and instrumental punishment (“Has stolen from you”). The second block focuses on the adolescents’ perception of the profile of the victim, obtaining three scores: fearful, trapped, and mistreated. Finally, the third block delves into the victimization relationship, addressing such aspects as the duration of the relationship, number of attempts to break up, etc. The match of this questionnaire to the study’s samples is evidenced by the reliability indices (Cronbach’s alpha) that were obtained, which ranged between 0.66 and 0.83 for the different violence sub-scales.
- *Mechanisms of Moral Disengagement Scale (MMDS)* [33]. This questionnaire has 32 items that allow one to obtain 8 partial scores which correspond to the 8 factors of moral disengagement: moral justification, euphemistic language, advantageous comparison, displacement of responsibility, diffusion of responsibility, distortion of consequences, attribution of blame, and dehumanization. The scale used is a Likert scale with 5 anchor points ranging from “Strongly disagree” to “Strongly agree”. The overall internal consistency of the test (Cronbach’s alpha) is 0.74, and, the reliability of the 8 mechanisms range between 0.72 and 0.81. Finally, these 8 mechanisms can be grouped into 4 dimensions or factors: behavioral locus (0.75), outcome locus (0.78), agency locus (0.79), and recipients locus (0.81).

### 2.3. Procedure

Prior to the distribution of the questionnaires to the adolescents, both the research objectives and the procedure, instruments and techniques used were checked and approved by the Bioethics and Biosafety Committee of University of Extremadura (Spain) (Ref. 18/2017).

Before starting with the distribution and administration of the questionnaires to the adolescents, the schools were approached prior to authorization from the Regional Educational Administration to whom the project was presented, and access to them during school hours was facilitated. Then, the Management Teams of these lower and upper secondary schools were invited to participate in the study, describing to them the objectives and purpose of the study and the use of the data. The written invitation was followed by personal telephone calls to coordinate the collection of data covering the different levels.

Once authorizations to enter the schools had been obtained, parental approval was requested (as the participants were minors) by means of a document describing the nature of the study and the mechanisms used to guarantee the anonymity and confidentiality of the responses. The letter was accompanied by an authorization form which the parents had to sign and send back to the school if they wanted their children to participate in the study. After obtaining the authorization, the questionnaires were distributed in hard copies and completed by the participants.

### 2.4. Analysis

Knowledge of the relationship between the variables through a correlation analysis facilitates the development of a theoretical model of moderated mediation. The direct relationship between perception and victimization was examined using linear regression. Acceptance and moral disengagement were examined as mediators between perception and victimization. Finally, the moderating effect of moral disengagement was tested. To analyze the mediation effect of acceptance in the relationship between the perception of dating violence and victimization, we applied the mediation test of Baron and Kenny [34]. This test requires there to be significant relationships between perception and victimization, perception and acceptance, and acceptance and victimization while controlling for the variable perception. Likewise, it requires there to be a significant coefficient of the indirect effects between perception and victimization via acceptance, a condition whose satisfaction was verified by the bias-corrected percentile bootstrap method. Finally, to determine whether this mediation process was moderated by the variable moral disengagement, the moderated mediation test of Hayes [35] was used.

## 3. Results

Firstly, the analysis of the results revealed that 17.28% of the 2577 adolescents who participated in the study had suffered some type of violence and classified themselves as victims in a relationship with their partner. Likewise, it was found that 15.33% (395 people) of these victims had suffered aggressions frequently, but the percentage of victimization fell to 1.59% (91 people) of the sample when the aggressions occurred almost always. Additionally, the results indicated that the most frequent types of violence were detachment (258 participants frequently: 65.31%, and 50 almost always: 54.9%), emotional punishment (188 frequently: 47.59%, and 42 almost always: 46.15%), and coercion (141 frequently: 35.69%, and 40 almost always: 43.95%). Finally, it stood out that some victims were subjected to more than one form of abuse at the same time (Table 1).

With respect to the perception that adolescents have of the violence they suffer within their dating relationship, it was found that 47 of them obtained scores that classified them with a fearful role, 235 victims were classified with a trapped role, and 17 with a mistreated role. Additionally, it was found that some of these adolescents obtained scores that grouped them under a double or triple role: 92 were classified as being at once fearful and trapped, 63 as fearful and mistreated, 80 as trapped and mistreated, and 50 as fearful, trapped, and mistreated.

The results of the correlation analysis allowed us to study the relationships between pairs of scores, taking into account victimization, gender, age, intensity, perception, acceptance of violence, and moral disengagement (Table 2).

It was found that “acceptance” of violence correlates with “age”—as age increases so does acceptance of violence. On the other hand, “perception” correlates with “intensity”, but the relationship is inverse – the stronger the intensity of violence, the weaker its perception. Also, as the “intensity” of the aggression suffered increases, the victims declare themselves to feel more “victimized” (mistreated, fearful, or trapped). The variable “perception” correlates with “acceptance” with the relationship being inverse—the stronger the perception of aggression, the lower the acceptance, i.e., as participants report feeling mistreated, fearful, or trapped, their level of acceptance of the violence decreases. Another significant correlation is found between “perception” and “moral disengagement”, finding that the greater the perception, the greater the use of disengagement mechanisms. Finally, there is a statistically significant correlation between “moral disengagement” and “acceptance” of violence, finding that the greater the acceptance, the greater the moral disengagement (Table 2).

**Figure 1:**
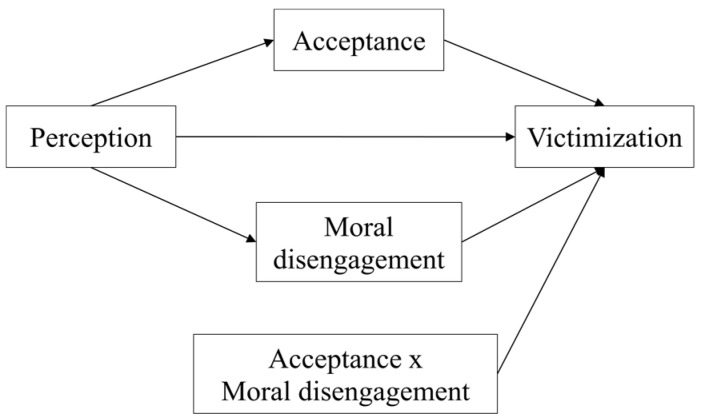
Theoretical model of moderated mediation.

After measuring the association of the variables through the correlation analysis, we constructed the moderated mediation model of the direct and indirect relationships between the variables included in this study of violence in teenage dating. Specifically, this model predicts victimization through the perception the victims have of violence, with this variable being mediated and moderated by moral disengagement and the acceptance of violence, as well as by the interaction between the two. For this, the theoretical model shown in Figure 1 was followed.

The analysis of the effects that the different variables of the study (gender, age, intensity, and perception) have on the two mediating and moderating variables “acceptance” and “moral disengagement” established Model 1. In this model, “perception of violence” negatively influences “acceptance” (*β* = −0.19, *p* < 0.05). Additionally, the “intensity of violence” has no statistically significant influence on “acceptance” (Model 1) (Table 3). These results indicate that the “perception” of violence is modified by the variable “acceptance”, implying that high levels of “acceptance” correspond to low levels of “perception” of violence.

Secondly, Model 2 was established, analyzing the influence the different variables have on the moderating variable “moral disengagement”. This model reflects the lack of any action of age or intensity of the violence on the moral disengagement of the victims. However, the variable “perception” is found to have a statistically significant influence on “moral disengagement” (*β* = 0.18; *p* < 0.05) (Model 2) (Table 3). According to these results, the “perception” of violence implies a greater use of “moral disengagement”.

Finally, Model 3 (Table 3) analyzes the impact that the different variables in the model have on “victimization”. First, there is the influence that “intensity” has on “victimization” (*β* = 0.17; *p* < 0.05), and similarly the “perception” of violence is also found to influence “victimization” (*β* = 0.40; *p* < 0.001). These two findings imply that both the “intensity” and the “perception” of the violence act on “victimization”, enhancing it as the levels of those two variables increase. Finally, it is found that the interaction of the variables “moral disengagement” and “acceptance” of violence have a statistically significant influence on “victimization” (*β* = 0.51; *p* < 0.001). In particular, the interaction between the two variables implies that a greater “acceptance” of the violence and the use of “moral disengagement” mechanisms on the part of the victims generates greater “victimization”.

With regard to direct effects, the results indicate that neither “gender” nor “age” have a direct influence on “victimization”. Furthermore, the variable “perception” is found to have a direct negative effect on “acceptance” (*β* = −0.19; *p* < 0.001), and a direct positive effect on “moral disengagement” (*β* = 0.17, *p* < 0.01) (Table 3). These findings indicate that the “perception” of violence causes less “acceptance” of the aggressions, but leads to greater use of the “moral disengagement” mechanisms.

With respect to the variable “victimization”, it is found that “acceptance” has a direct negative effect on it (*β* = −0.19; *p* < 0.001), while the direct effect of “moral disengagement” is positive (*β* = 0.17, *p* < 0.01) (Table 4). These facts indicate that “acceptance” of the violence implies lesser “victimization”, and that high levels of “moral disengagement” imply greater “victimization”.

Finally, there is a positive direct effect of “perception” on “victimization” (*β* = 0.41; *p* < 0.001) (Table 4), thus revealing that the greater the “perception”, the greater the “victimization”.

**Figure 2:**
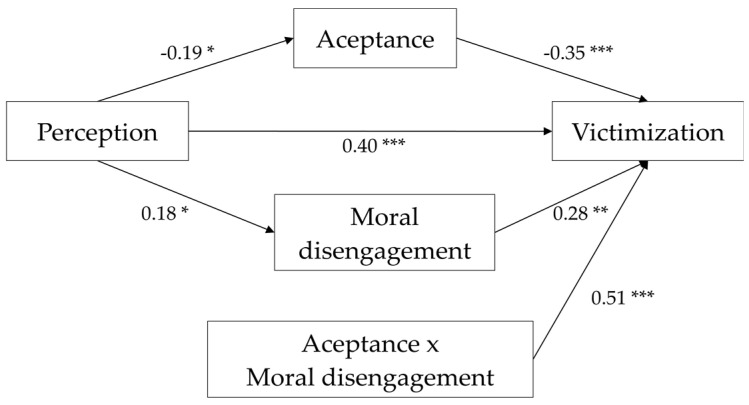
Mediation model results; * *p* < 0.05; ** *p* < 0.01; *** *p* < 0.001.

Together, the above results reveal a network of relationships between the variables studied that is summarized graphically in Figure 2. This indicates the influence that the perception of violence has on victimization, and that this influence is mediated and moderated directly and indirectly by the variables acceptance of violence and moral disengagement, as well as by the interaction between the two.

## 4. Discussion

With the intention of shedding more light on the understanding of the victimization process and the causes of its appearance and persistence, this study has revealed the existence of a complex network of relationships affecting this process in teenage dating relationships. The results indicate that aspects such as age, gender, the perception that adolescents have of their role in victimization, acceptance of the abuses, and moral disengagement have a decisive influence on victimization. That impact is established through relationships that may be positive, as in the case of moral disengagement, or negative, as in the case of the perception of violence. Finally, the acceptance of violence and the mechanisms of moral disengagement become variables that mediate and moderate the relationship between the victims’ perception of aggression and the process of victimization. The relationship of all these variables indicates the importance of adopting an ecological approach that allows addressing violence and victimization at both the individual and relational, organizational and community levels [36].

Thus, focusing attention on the factors that influence the way adolescents value abuses, it was found that variables such as gender, age, and perception of violence significantly influence the degree to which victims accept this type of behavior (Model 1). With regard to gender, it was found that girls are victimized more than boys. A possible explanation for this fact has been given by some authors who link victimization with the attachment system that the two genders develop. In this sense, Furman and Simon [37] note that men tend to show greater avoidant attachment and lesser anxious attachment. Likewise, the anxious attachment that some women have has been related to greater victimization [38]. However, other authors have related victimization to aspects that are more interconnected with the role that society gives to men and women. In this sense, Meza [39] points out that Spain is still to a certain extent a traditional country where boys tend to assume roles in which active and dominant attitudes predominate, while the socialization of girls implies that they tend to be characterized as being more sensitive and passive. Similarly, Viki, Abrams, and Hutchison [40] note that adolescent girls tend to be characterized as weak persons who need to be protected by their partners. This belief would not only devalue women, but would cause aspects of the phenomenon of dating violence such as control by the partner not to be frowned upon by society, since these aspects are consistent with the aforementioned gender stereotypes [16].

The lesser victimization found in boys could be related to other aspects such as those noted by Molidor and Tolman [41], who indicate that when boys are victimized, they are less marked by the abuse than girls, and experience fewer negative consequences. Therefore, the suffering would be less, as also would be their perception of the role of victimization. In this sense, it is important also to note that, according to authors such as Karlsson, Temple, Weston, and Lee [42], boys are more accepting than girls of teenage dating violence, while society as a whole is more accepting of dating violence perpetrated by girls than by boys. These facts would imply that boys reject violence to a lesser extent than girls, at the same time as tending to justify it [43].

The variable age was found to be related to the acceptance of violence, although not to its frequency. In this sense, the present study found that the lowest number of victims was in the group of older adolescents, i.e., those between the ages of 16 and 18, with the youngest group being the ones who accepted violence to the greatest extent. The link of adolescence with the belief in romantic love and the soulmate could be one of the explanations that would help to understand why it is precisely the younger adolescents who are most accepting of violence. In these early stages, the adolescents’ own inexperience would mark their idealization of both their partner and the relationship itself, and all this would derive into the development of maladaptive behavior [44]. Indeed, some research has linked the development of denial and non-confrontational behavior in adolescent girls to the non-recognition of a conflictive situation [45].

In contrast, it can also be seen how the results of the present study reveal that age is related to the intensity of violence, with this being greater in the adolescents closer to adulthood. This relationship can probably be understood by taking into account the explanations proposed by Menesini and Nocentini [46] who indicated that consolidation of the dating relationship entails a greater tendency towards conflict. At the same time, such conflict would imply violence that is more intense [47]. Time would then act as a disinhibitor of the behavior, and it can be said that the longer the relationship, the greater the violence [48].

With regard to the perception of abusive behavior in dating relationships, the results of the study reveal that this construct significantly influences victimization in adolescents. Specifically, it was found that there is an increase in victimization due to the absence of the perception of violence. This finding is especially serious since, if adolescents do not perceive themselves as victims, the task of detection and subsequent intervention becomes even harder. First, this link between the low perception of violence and victimization could again be explained by the typical view during adolescence of romantic love as a form of idealization of relationships. It is relatively common to find that the first conflicts, or the first abuses, far from leading to breakup, become what both members of the couple understand as being “proofs of love” [15]. This type of conduct is then not only justified, but also routinely denied, by the victims, especially when it occurs sporadically [49].

Second, in addition to the predominance of romantic love during the adolescent stage, the low perception of violence and the high acceptance of aggression at these ages could be explained through emotional-type variables. Thus, emotional dependence could be another contributing factor to the invisibility and denial of abuse in teenage dating relationships, leading to the victim having a low perception of their own victimization. In this regard, García [50] found that adolescents under the age of 18 consider giving themselves to the other person as being of vital importance in dating relationships. This gift of themselves includes both pleasant and painful aspects, and implies the beginning of a relationship of dependency which, on many occasions, they are unaware of. For the victims, enduring humiliation and abuse becomes part of the relationship to such an extent that the dependent partner is willing to do anything to maintain the relationship [51]. In this sense, the idealization of love could lay the foundations for emotional dependence, combining both aspects and giving rise to the acceptance of abuse.

And third, one must highlight the negative relationship found between intensity of the violence and acceptance of this type of behavior. This finding indicates that, as the intensity of the violence increases, adolescents tend to accept this type of behavior less and vice versa. These results point in the same direction as those of previous studies indicating that people who are aware of their victimization, and perceive themselves as being mistreated in their dating relationship, are precisely those with the greatest experience of victimization [32].

Analysis of the results reveals another fact to add to the already complex network of relationships established between the different variables considered in the phenomenon of teenage dating violence. Specifically, there is a strong relationship between perception of violence and moral disengagement (Model 2), with the latter variable also being a mediating factor that modifies the perception of victimization, i.e., when violence is perceived, mechanisms of moral disengagement come into play, making those who are in an abusive relationship capable of minimizing the situation of aggression.

Although it is true that moral disengagement on the part of the aggressors as a variable involved in the perpetration of violent behavior has been a well-established aspect of research [52], the point of view of the victims has as yet been far less often the subject of consideration. There has, however, been some research carried out in recent years, with the results reported being similar to those of the present study. Thus, some authors indicate, within the contexts of school bullying, that victimized adolescents tend to develop a higher level of moral disengagement that allows them to justify, reconstruct, or rationalize violent behavior [25]. Cuadrado & Fernández [30] also describe the mechanisms of moral disengagement as playing a mediating role in the relationship created between the perception of cyberbullying and cybervictimization. In particular, the victims’ perception of the aggressions causes them to activate the mechanisms responsible for distorting the consequences of that victimization, for example.

To the above results should be added the finding that moral disengagement is not only directly involved in victimization, but also through its interaction with the acceptance of violence, with both factors taking on moderating and mediating roles that jointly affect the victimization process (Model 3). This is found to occur regardless of the gender of the victims. This means that, within a cognitive focus on morality, moral disengagement could interact with different contextual variables (behavior, emotions, etc.), with it being possible to predict different moral outcomes [53]. With this, it could be said that victims would use moral disengagement mechanisms as one more strategy with which to minimize the violence perpetrated in this type of abusive relationship. In this sense, the use of these mechanisms allows cognitive reconstruction of the aggressions so that they seem to be less harmful or even not harmful at all [54]. Such use would therefore be the key for victimized adolescents to accept aggressions without perceiving themselves as victims. With this, the victims’ moral disengagement fulfils a function that is similar to the one it has for the aggressors who are capable of perpetrating morally reprehensible behavior without self-condemnation of their actions [19].

Therefore, in accordance with some authors, it can be said that moral disengagement becomes a changing social cognitive factor that can be influenced by external factors such as the peer group or family environment, among others [53,55]. Once activated, these moral disengagement mechanisms moderate the influence that other variables have on the victimization process. These results would support a relationship that has been noted before in studies focused on the figure of the aggressor. Thus, some authors indicate that adolescents who had experienced higher levels of abuse during their lives were precisely those who tended to score higher in moral disengagement [25]. Others researchers describe the use of moral disengagement as being habitual among adolescents of ages from 16 to 18, with the use of moral justification and dehumanization being common [56].

Similarly, the present study reveals that adolescents who are mistreated in their dating relationship use moral disengagement as a way to minimize, normalize, and accept the aggression. These findings are supported by the results of other studies carried out in diverse contexts, such as bullying or cyberbullying. Hence, it seems that the victimization process follows common shared patterns regardless of the context in which it takes place. Thus, with the formulation of his cognitive theory, Bandura [57] already noted how the use of these mechanisms allows victims to reduce the tension they experience when other people do not respect their moral values, and they are unable to face and stop the situation. Given these facts, Bandura explains that victims tend to distort the abuse and aggression.

More recently, other workers have found that victims use moral disengagement as a way to justify not only their inaction when faced with aggression, but even the violence itself they have suffered [28,29]. Likewise, the fact that victims also use moral disengagement would explain the difficulties that some authors have encountered in breaking with the normalization of violence that can remain stable for periods of up to six years [58]. According to authors such as de la Villa, García, Cuetos, and Sirvent [44], this stability is transferred to adulthood, with the victimized adolescents becoming victimized adults, thereby causing any intervention to become increasingly difficult.

## 5. Limitations

This work has some limitations. First, it was a cross-sectional study, so that there must be caution in making any generalization of the results or in determining any causal and predictive relationships. Second, the analysis did not take age into account as a variable. Although the ages of the participants cover an interval that is not very broad (14–18 years old), the evolutionary moment at which these adolescents find themselves may have had some influence on the results. Moral disengagement is more settled at the end of the adolescence than in the early or mid-adolescence, and this may affect the use of the mechanism of moral disengagement.

These limitations serve to orient the consideration of new research to gain deeper knowledge of the process of dating violence and victimization. A longitudinal approach may cover the different evolutionary moment of the adolescence.

## 6. Conclusions

Dating violence has been the subject of study recently in different countries. However, the usual approach has been to take as referent the aggressors’ point of view with the aim of seeking explanations underlying their behavior. One of the major contributions of the present study has been to shift the attention to the victims, in an attempt to understand why these adolescents tend to normalize the situation and accept abusive behavior without being aware of their own victimization. The study of the interrelationships between the victims’ moral disengagement and their acceptance of violence reveals that both variables take on a moderating and mediating role with respect to the other constructs involved in the victimization process that arises in the phenomenon of teenage dating violence. In this way, the influence of both variables takes place by way of moderating and mediating, directly and indirectly, the adolescents’ perception of the abuse and their acceptance of it, and their assumption of their consequent role as victim.

Overall, these findings open the door to the consideration of more complex explanations instead of the search for simple or one-way relationships, with a view to prevention through family, formal, and socio-community education.

## Figures and Tables

**Table 1:** Frequency and modalities of victimization.

	Frequently	Usually
Victims	395 (15.33%)	91 (1.59%)
Detachment	258 (65.31%)	50 (54.9%)
Humiliation	82 (20.75%)	10 (10.98%)
Sexual	73 (18.48%)	20 (21.97%)
Coercion	141 (35.69%)	40 (43.95%)
Physical	34 (8.60%)	9 (9.89%)
Gender-Based	90 (22.78%)	19 (20.87%)
Emotional Punishment	188 (47.59%)	42 (46.15%)
Instrumental	42 (10.63%)	6 (6.59%)

**Table 2:** Correlations between the variables studied.

Variables	1	2	3	4	5	6	7
Victimization	-						
Gender	0.16	-					
Age	0.15	0.04	-				
Intensity	0.22 *	0.11	0.09	-			
Perception	0.48 ***	0.19 *	0.14	0.21 *	-		
Acceptance	−0.44 ***	0.31 ***	0.20 *	−0.19 *	−0.21 *	-	
Moral disengagement	0.33 **	0.26 **	0.12	0.15	0.26 **	0.61 ***	-

* *p* < 0.05; ** *p* < 0.01; *** *p* < 0.001.

**Table 3:** Moderated mediation effect on victimization.

Variables	Model 1 Mediator: Acceptance		Model 2 Mediator: Moral Disengagement		Model 3 Dependent Variable: Victimization	
	Β	SE	*β*	SE	Β	SE
Gender	0.24 **	0.04	0.20 **	0.03	0.06	0.03
Age	0.17 *	0.04	0.09	0.04	0.05	0.04
Intensity	−0.16	0.03	0.11	0.03	0.17 *	0.02
Perception	−0.19 *	0.04	0.18 *	0.03	0.40 ***	0.04
Acceptance					−0.35 ***	0.06
Moral disengagement					0.28 **	0.03
Acceptance x Moral disengagement					0.51 ***	0.05
*R* ^2^	0.15		0.09		0.19	
*Adj. R* ^2^	0.12		0.07		0.16	
*F*	26.14		12.35		17.83	

* *p* < 0.05; ** *p* < 0.01; *** *p* < 0.001; B: beta; SE: typical error of beta.

**Table 4:** Direct effects on victimization.

	*β*	SE
Direct effects of IV on mediators Perception →Acceptance Perception → Moral disengagement	−0.19 *** 0.17 **	0.04 0.02
Direct effects of mediators on DV Acceptance → Victimization Moral disengagement → Victimization	−0.39 *** 0.28 ***	0.05 0.05
Direct Effects of IV on DV Perception → Victimization	0.41 ***	0.04
*R* ^2^	0.14	
*Adj. R* ^2^	0.11	
*F*	18.36	

Note: *n* = 2577—Gender, age, and intensity were controlled; ** *p* < 0.01; *** *p* < 0.001.
